# Patricia Keely (1963-2017)

**DOI:** 10.1242/bio.030098

**Published:** 2017-10-15

**Authors:** Jordan W. Raff

**Affiliations:** Editor in Chief Biology Open (bio@biologists.com)

All of us at Biology Open were very sad to learn of the death of Patricia Keely, one of our longest-serving Editors. Patricia joined the team upon the launch of The Company of Biologists' newest journal in 2011, demonstrating her strong support for not-for-profit scientific publishing by taking a chance on a fledgling journal that had no previous track record. She brought her expertise to bear on many articles over the years, dedicating a considerable amount of time and effort and exercising her judgement with great wisdom. Although her illness meant that she was unable to attend our most recent Editor meeting, I was in contact with her during her treatment and she remained remarkably upbeat and enthusiastic about potential new treatments, despite her deep understanding of how serious the situation was and how poor her prognosis. Reading the many tributes to her has only impressed upon us the considerable legacy she leaves (http://keelylab.org/in-memoriam/; www.med.wisc.edu/news-events/remembering-patricia-keely-scientist-mentor-friend-and-inspiration/51087). We send our condolences to her friends, family and colleagues.

